# Characteristics associated with progression in patients with of nontuberculous mycobacterial lung disease : a prospective cohort study

**DOI:** 10.1186/s12890-016-0349-3

**Published:** 2017-01-05

**Authors:** Soo Jung Kim, Soon Ho Yoon, Sun Mi Choi, Jinwoo Lee, Chang-Hoon Lee, Sung Koo Han, Jae-Joon Yim

**Affiliations:** 1 Division of Pulmonary and Critical Care Medicine, Department of Internal Medicine, School of Medicine, Ewha Womans University, Seoul, Republic of Korea; 2 Department of Radiology, Seoul National University College of Medicine, Seoul, Republic of Korea; 3 Division of Pulmonary and Critical Care Medicine, Department of Internal Medicine, Seoul National University College of Medicine, 101 Daehak-Ro, Jongno-Gu Seoul, 110-744 Republic of Korea

**Keywords:** Abdominal fat, Anthropometry, Body composition, Nontuberculous mycobacteria

## Abstract

**Background:**

Patients with distinctive morphotype were more susceptible to nontuberculous mycobacterial lung disease (NTM-LD). However, little is known about the association between body morphotype and progression of NTM-LD. The aim of this study was to elucidate predictors of NTM-LD progression, focusing on body morphotype and composition.

**Methods:**

Data from patients with NTM-LD who participated in NTM cohort which started in 1 July 2011 were analyzed. Patients with more than 6 months of follow up were included for analysis. NTM-LD progression was defined as clinician-initiated anti-NTM treatment, based on symptomatic and radiologic aggravation. Body morphotype and composition was measured at entry to the cohort using bioelectrical impedance analysis.

**Results:**

NTM-LD progressed in 47 out of 150 patients with more than 6 months of follow up. Patients with middle (adjusted hazard ratio [aHR], 2.758; 95% confidence interval [CI], 1.112–6.843) or lowest tertile (aHR, 3.084; 95% CI, 1.241–7.668) of abdominal fat ratio had a higher risk of disease progression compared with the highest tertile. Other predictors for disease progression were presence of cavity on chest computed tomography (aHR, 4.577; 95% CI, 2.364–8.861), and serum albumin level <3.5 g/dL (aHR, 12.943; 95% CI, 2.588–64.718).

**Conclusions:**

Progression of NTM-LD is associated with body composition. Lower abdominal fat ratio is an independent predictor of NTM-LD progression.

**Trial registration:**

ClinicalTrials.gov, NCT01616745 Registered 25 March 2012

## Background

Nontuberculous mycobacterial lung disease (NTM-LD) has been increasing prevalence in many part of the world [1–4]. Unlike *Mycobacterium tuberculosis* (TB), the diagnosis of NTM-LD does not necessitate initiation of therapy, and treatment should be decided based on the potential risks and benefits of therapy for individual patients [1]. Previous reports have indicated that about half of patients with *Mycobacterium avium* complex (MAC) lung disease required treatment eventually [5, 6].

Given the difficulties in predicting which patients with NTM-LD will experience disease progression, an observation period is usually needed to decide whether or not to initiate treatment [7, 8]. In MAC lung disease, the presence of cavities or consolidation on initial chest computed tomography (CT) was suggested to be associated with disease progression and treatment [5]. Thinner chest subcutaneous fat thickness on chest CT or higher number of involved lung segments were also suggested as risk factors for radiologic deterioration [9]. Another study demonstrated that patients with *M. intracellulare* lung disease, positive sputum smears, or fibrocavitary form were more likely to receive anti-NTM treatment [10].

It has been shown that patients with a distinctive body morphotype are more susceptible to NTM-LD; patients with pulmonary NTM infection were taller and leaner than controls, while skin fold and circumference measurements were also significantly leaner [11]. In addition, lower body mass index (BMI) [12, 13], lower percent body fat and total body fat [12], and low subcutaneous fat [14] have been reported among patients with NTM-LD.

However, little is known about the association between body morphotype or composition and the progression of NTM-LD. The objective of the present study was to elucidate the impact of body morphotype and composition on the progression of NTM-LD through analysis of a prospective cohort of patients with NTM-LD.

## Methods

### Participants

Patients with NTM-LD who participated in a previously described ongoing observational, prospective study [13], which begun on 1 July 2011 at Seoul National University Hospital were included and analyzed. Study inclusion criteria were: aged between 20 and 80 years, NTM-LD that fulfilled the diagnostic criteria reported in the 2007 American Thoracic Society/Infectious Diseases Society of America guidelines [1]. Patients previously treated for NTM-LD were excluded from this study. The clinical trial registration number for the study is NCT01616745 (www.ClinicalTrials.gov). All patients provided written informed consent before enrollment. Retrieval of data for the current study was performed on 1 April 2015. Patients followed for fewer than 6 months, and patients without follow-up CT scans, were excluded.

### Study design

At study enrollment of the participants, demographic, clinical, laboratory, and radiographic data, and body morphotype characteristics were collected and analyzed. Body morphotype and composition including height, weight, BMI, total body muscle, total body fat, percentage body fat, abdominal fat ratio, waist circumference, and hip circumference were measured using InBody 720 (Biospace Co, Seoul, South Korea) which utilized the bioelectrical impedance analysis method. This body composition analyzer assumes the body comprises five cylinders: four limbs and the trunk, and measures impedance of these parts separately. This method provides segmental measurement of body water and fat-free mass. The abdominal fat ratio is the ratio of fat stored in the waist to fat stored in the hips. Each morphometric measurement was categorized according to tertiles for analysis. The levels of adipokines were also measured at study entry. Serum level of leptin was measured using radioimmunoassay (RIA) kit (LINCO Research, Inc., U.S.A) and serum level of adiponectin was measured using an enzyme-linked immunosorbent assay (ELISA) kit (Biovendor, Brno, Czech Republic).

All patients had a chest CT scan at study entry. CT scans were evaluated by a board-certified radiologist using a previously published scoring system [15]. A total of six lung lobes (right upper lobe, right middle lobe, right lower lobe, upper division of the left upper lobe, lingular division of the left upper lobe, and left lower lobe) in each patient were assessed for the presence of lung parenchymal abnormalities. Scores were assigned by considering the presence, severity, and extent of bronchiectasis (maximum score, 9), cellular bronchiolitis (maximum score, 6), cavity (maximum score, 9), nodules (maximum score, 3), and consolidation (maximum score, 3).

Progression of NTM-LD was defined as initiation of anti-NTM treatment by the duty physician based on symptomatic (*e.g.*, increased amount of sputum) or radiographic aggravation (*e.g.*, cavity formation). Four of the authors (Choi, J Lee, CH Lee, and Yim) participated in the care of and discussed the treatment initiation for each patient. Patients who refused treatment despite the physician’s recommendation were also classified as having disease progression.

### Mycobacterial culture and Species identification of NTM

Acid-fast bacilli (AFB) smears and mycobacterial cultures were performed as recommended in the standard guidelines [16]. All cultures were grown in both solid Ogawa media and the BACTEC MGIT 960 system. NTM species were identified using sequence analysis of the 16S rRNA gene by the algorithm descried in the Clinical and Laboratory Standards Institute (CLSI) guidelines [17]. Sequencing of the *rpoB* and *tuf* genes was performed for further identification. In particular, differentiation between *M. abscessus* and *M. massiliense* was based on analysis of the *rpoB* gene sequence [18, 19]. Taxonomy of subspecies of the *M. abscessus* complex was described based on recent suggestions [20]. Patients were considered to have a mixed NTM species infection if NTM species other than the original one were isolated at least twice during the follow-up period until the start of treatment.

### Statistical analysis

Baseline characteristics at the study initiation date (including age, sex, comorbidities, symptoms at initial visit, abnormal breathing sound, anxiety, depression, St. George’s Respiratory Questionnaire (SGRQ) score, chest CT score, presence of cavities, pulmonary function, cholesterol and albumin levels, body morphotype and composition) for patients with and without disease progression were summarized using descriptive statistics such as proportion, median and interquartile range (IQR). Continuous variables were categorized into tertiles (for example, body morphotype and composition such as abdominal fat ratio) or appropriate categorical variables based on normal range (for example, albumin levels).

Cumulative disease progression was estimated using the Kaplan-Meier method. A cox-proportional hazard regression model was used to find predictors of disease progression. Covariates with a *p*- value <0.2 were used in multivariate analyses. Multivariate analyses were constructed using the stepwise backward elimination method, which accounts for collinearity problems. All statistical analyses were performed using SPSS 18.0 (SPSS Inc., Chicago, IL, USA).

## Results

### Baseline patient characteristics

Since July 1st 2011, 207 patients with NTM-LD have been enrolled into the original study. 51 patients without follow up CT scans of the chest and six patients followed up shorter than 6 months were excluded. Finally, data for 150 patients were analyzed in the current study. The median age of the 150 patients was 64 years, 92 patients (61.3%) were female, and 110 (73.3%) were never smokers (Table 1). Median height was 160.4 cm, median weight was 54.1 kg, and median BMI was 21.1 kg/m^2^. Median body fat and abdominal fat ratio were 13.5 kg and 0.86 (Table 2).

**Table 1:** Baseline characteristics of 150 participants with nontuberculous mycobacterial lung disease at study entry

	Total patients (*N* = 150)	Progressed (*N* = 47)	Not progressed (*N* = 103)	*p*-value
Age, years	64 [55–72]	62 [54–68]	66 [56–74]	0.030
Sex, female	92 (61.3)	32 (68.1)	60 (58.3)	0.251
Smoking				0.160
Ever-smoker	40 (26.6)	9 (19.2)	31 (30.1)	
Never-smoker	110 (73.3)	38 (80.9)	72 (69.9)	
Past medical history				
Tuberculosis	55 (36.9)	20 (43.5)	35 (34.0)	0.267
Measles	29 (19.3)	7 (14.9)	22 (21.4)	0.352
Pertussis	7 (4.7)	3 (6.4)	4 (3.9)	0.678
Comorbidity				
COPD	27 (18.8)	6 (12.8)	21 (21.6)	0.200
Asthma	4 (2.8)	0 (0)	4 (4.1)	0.304
Diabetes	14 (9.3)	5 (10.6)	9 (8.7)	0.765
Malignancy	20 (13.3)	4 (8.5)	16 (15.5)	0.241
Symptom at initial visit				
Cough	61 (40.7)	23 (48.9)	38 (36.9)	0.164
Sputum	108 (72.0)	35 (74.5)	73 (70.9)	0.649
Dyspnea^a^	15 (10.0)	3 (6.4)	12 (11.3)	0.392
Hemoptysis	29 (19.3)	13 (27.7)	16 (15.5)	0.081
Fever	19 (12.7)	5 (10.6)	14 (13.6)	0.614
Weight loss	17 (11.3)	8 (17.0)	9 (8.7)	0.138
Abnormal breath sounds	20 (13.3)	9 (19.1)	11 (10.7)	0.157
Crackles	11 (7.3)	4 (8.5)	7 (6.8)	0.741
Wheezing	13 (8.7)	7 (14.9)	6 (5.8)	0.113
Anxiety^b^	29 (19.3)	11 (23.4)	18 (17.5)	0.394
Depression^b^	41 (27.3)	16 (34.0)	25 (24.3)	0.213
SGRQ	21.2 [11.3–35.2]	24.4 [12.7–44.1]	19.8 [10.3–30.6]	0.011
Radiologic features				0.599
Nodular bronchiectatic disease	135 (90)	41 (30.4)	94 (69.6)	
Fibrocavitatary disease	14 (9.3)	6 (42.9)	8 (57.1)	
Unclassifiable	1 (0.7)	0 (0)	1 (1.0)	
Chest CT score^c^	10 [7–13]	13 [8–15]	9 [7–12]	<0.001
Radiologic deterioration^d^	58/130 (44.6)	21/27 (77.8)	37/103 (35.9)	<0.001
Presence of cavity	37 (24.7)	24 (51.1)	13 (12.6)	<0.001
Pulmonary function				
FVC, L	2.83 [2.32–3.42]	2.54 [2.23–3.16]	2.97 [2.36–3.48]	0.032
FVC, %	93 [81–101]	88 [79–95]	94 [82–106]	0.005
FEV_1_, L	2.09 [1.76–2.48]	2.11 [1.77–2.38]	2.08 [1.73–2.51]	0.532
FEV_1_, %	97 [82–110]	93 [82–105]	99 [81–113]	0.205
DLCO, %	93 [80–106]	93 [80–104]	93 [81–107]	0.998
Cholesterol, mg/dL	182 [161–204]	178 [146–202]	183 [167–205]	0.229
Albumin, g/dL	4.3 [4.0–4.4]	4.2 [4.0–4.4]	4.3 [4.1–4.5]	0.031

Data are presented as median [interquartile range] or proportion (%)

*COPD*, chronic obstructive pulmonary disease; *SGRQ*, St. George’s Respiratory Questionnaire; *CT*, computed tomography; *FVC*, forced vital capacity; *FEV*_*1*_, forced expiratory volume in 1 s; *DLCO*, diffusing capacity for carbon monoxide

^a^Dyspnea was defined as modified Medical Research Council score ≥ 2

^b^Hospital Anxiety and Depression Scale (HADS), same or > 8

^c^Scores were given by considering the severity of bronchiectasis (maximum score, 9), cellular bronchiolitis (maximum score, 6), cavity (maximum score, 9), nodules (maximum score, 3) and consolidation (maximum score, 3). Maximum score possible was 30

^d^Radiologic deterioration was defined as a one-point increment in the CT score on follow-up CT scans of the chest. Follow-up CT scans taken before the initiation of treatment were available in 27 out of 47 patients in whom nontuberculous mycobacterial lung disease progressed

**Table 2:** Body morphotype and composition of 150 participants with nontuberculous mycobacterial lung disease at study entry

	Total patients (*N* = 150)	Progressed (*N* = 47)	Not progressed (*N* = 103)	*p*-value
Height, cm	160.4 [155.0–167.2]	159.0 [154.0–167.1]	162.0 [156.4–167.3]	0.227
Weight, kg	54.1 [49.1–60.0]	51.0 [45.0–57.0]	55.5 [50.0–61.8]	0.002
BMI, kg/m^2^	21.1 [19.3–22.8]	19.9 [18.5–21.7]	21.6 [19.8–23.2]	0.003
Waist, cm	76.0 [70.0–83.0]	72.0 [67.0–82.0]	77.0 [72.0–85.0]	0.008
Hip, cm	92.0 [89.0–97.0]	90.0 [88.0–92.0]	93.0 [90.0–97.0]	0.004
Skeletal muscle mass, kg	21.3 [19.0–24.4]	19.9 [17.3–22.5]	21.9 [19.6–24.7]	0.015
Body fat, kg	13.5 [10.1–17.4]	12.0 [9.1–13.8]	14.9 [11.0–18.5]	0.003
Body fat percentage, %	25.2 [20.2–30.4]	23.8 [18.6–26.0]	27.1 [20.4–31.0]	0.044
Abdominal fat ratio	0.86 [0.83–0.91]	0.85 [0.81–0.87]	0.87 [0.83–0.92]	0.004

Data are presented as median [interquartile range]

BMI, body mass index

### Nontuberculous mycobacterial species isolated from participants

MAC was most commonly isolated from participants: *M. avium* (54 patients, 36.0%), *M. intracellulare* (40 patients, 26.7%), *M. chimaera* (1 patient, 0.7%), *M. yongonense* (1 patient, 0.7%), *M. avium* and *M. intracellulare* (10 patients, 6.7%), *M. avium* and *M. chimaera* (1 patient, 0.7%), and MAC and other species (2 patients, 1.3%). *M. abscessus* complex (MABC) was the second most common isolate: *M. abscessus* subsp. *abscessus* (14 patients, 9.3%), *M. abscessus* subsp. *massiliense* (12 patients, 8.0%), mix of both species (4 patients, 2.7%), and MABC and other species (1 patient, 0.7%) (Table 3).

**Table 3:** Mycobacterial species isolated from 150 participants

Species	Total patients (*N* = 150)	Progressed (*N* = 47)	Not progressed (*N* = 103)	*p*-value
*Mycobacterium avium* complex (MAC)				
*M. avium*	54 (36.0)	15 (31.9)	39 (37.9)	0.481
*M. intracellulare*	40 (26.7)	17 (36.2)	23 (22.3)	0.075
*M. chimaera*	1 (0.7)	0 (0)	1 (1.0)	1.000
*M. yongonense*	1 (0.7)	0 (0)	1 (1.0)	1.000
*M. avium* and *M. intracellulare*	10 (6.7)	3 (6.4)	7 (6.8)	1.000
*M. avium* and *M. chimaera*	1 (0.7)	1 (2.1)	0 (0)	0.313
MAC and others^a^	2 (1.3)	1 (2.1)	1 (1.0)	0.530
*M. abscessus* complex (MABC)				
*M. abscessus* subsp. *abscessus*	14 (9.3)	4 (8.5)	10 (9.7)	1.000
*M. abscessus* subsp. *massiliense*	12 (8.0)	3 (6.4)	9 (8.7)	0.754
*M. abscessus* subsp. *abscessus* and *massiliense*	4 (2.7)	1 (2.1)	3 (2.9)	1.000
MABC and others^b^	1 (0.7)	0 (0)	1 (1.0)	1.000
MAC and MABC	7 (4.7)	2 (4.3)	5 (4.9)	1.000
*M. fortuitum*	2 (1.3)	0 (0)	2 (1.9)	1.000
*M. kyorinense*	1 (0.7)	0 (0)	1 (1.0)	1.000

Data presented as n (%)

^a^Others: *M. fortuitum* (1 patient), *M. kansasii* (1 patient)

^b^Others: *M. conceptionense* (1 patient)

MAC, *Mycobacterium avium* complex; MABC, *Mycobacterium abscessus* complex

### Progression of nontuberculous mycobacterial lung disease

**Fig. 1:**
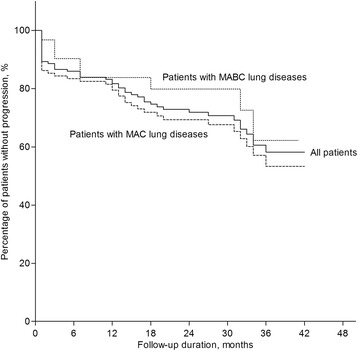
Disease progression among 150 patients with nontuberculous mycobacterial lung disease No difference was identified in progression rate between patients with *M avium* complex (MAC) and *M. abscessus* complex (MABC) (P = 0.323)

Median duration of follow-up of the 150 study participants was 28 months (IQR: 18 –36 months). During the study period, NTM-LD progressed in 47 of 150 patients. 1- and 2-year disease progression rates were 18.2 and 28.1%, respectively (Fig. 1). Follow-up CT scans taken before the initiation of treatment were available in 27 of 47 patients in whom NTM lung disease progressed. The proportion of patients with radiographic worsening (defined as a one-point increment in the CT score on follow-up) was higher in the group that progressed than in the group that did not progress (77.8 *vs.* 35.9%, *P* < 0.001) (Table 1).

### Predictors of disease progression

Patients with progression of NTM-LD were younger (62.0 *vs.* 66.0 years, *p* = 0.030), had higher SGRQ (24.4 *vs.* 19.8, *p* = 0.011) and chest CT (13 *vs.* 9, *p* < 0.001) scores, and lower FVC (88 *vs.* 94%, *p* = 0.005) and albumin levels (4.2 *vs.* 4.3 g/dL, *p* = 0.031), compared with those without progression (Table 1).

Univariate analysis was performed to find predictors of disease progression. The lowest tertiles of weight, BMI, waist and hip circumference, skeletal muscle mass, body fat, and body fat percentage or abdominal fat ratio, were related to progression of NTM-LD (Table 4). Additionally, higher chest CT score, presence of cavities, serum albumin level < 3.5 g/dL, and FVC < 80% were also associated with disease progression.

**Table 4:** Predictors of disease progression: univariate analysis

	Unadjusted HR	95% CI	*p*-value
Age, years			
Q1 [<55, *n* = 31]	Ref.		
Q2 [55– 64, *n* = 40]	0.990	0.463–2.160	0.979
Q3 [65–72, *n* = 36]	1.052	0.480–2.309	0.899
Q4 [>72, *n* = 43]	0.429	0.169–1.089	0.075
Sex			
Male	Ref.		
Female	1.351	0.731–2.496	0.337
SGRQ			
< 25	Ref.		
≥ 25	1.562	0.875–2.789	0.132
Radiologic feature			
Nodular bronchiectatic disease	1		
Fibrocavitary disease	1.740	0.737–4.108	0.206
Chest CT score			
X	Ref.		
X + 1	1.211	1.123–1.306	<0.001
Presence of cavity			
Absent	Ref.		
Present	3.750	2.086–6.739	<0.001
Cholesterol, mg/dL			
≥ 200	Ref.		
< 200	1.263	0.673–2.371	0.467
Albumin, g/dL			
≥ 3.5	Ref.		
< 3.5	8.942	3.097–25.818	<0.001
FVC, %			
≥ 80	Ref.		
< 80	1.974	1.049–3.715	0.035
FEV_1_			
≥ 80	Ref.		
< 80	1.102	0.546–2.226	0.786
Height, cm			
High [>165, *n* = 48]	Ref.		
Middle [157–165, *n* = 55]	1.126	0.547–2.319	0.748
Low [<157, *n* = 47]	1.243	0.604–2.562	0.555
Weight, kg			
High [>59, *n* = 49]	Ref.		
Middle [50–59, *n* = 55]	1.935	0.861–4.347	0.110
Low [<50, *n* = 46]	3.146	1.437–6.885	0.004
BMI, kg/m^2^			
High [>22, *n* = 52]	Ref.		
Middle [20–22, *n* = 44]	1.619	0.669–3.752	0.261
Low [<20, *n* = 54]	3.211	1.538–6.705	0.002
Waist, cm			
High [>80, *n* = 50]	Ref.		
Middle [72–80, *n* = 41]	1.082	0.467–2.507	0.853
Low [<72, *n* = 37]	2.183	1.041–4.575	0.039
Hip, cm			
High [>95, *n* = 45]	Ref.		
Middle [90–95, *n* = 48]	2.062	0.890–4.780	0.092
Low [<90, *n* = 35]	2.485	1.041–5.929	0.040
Skeletal muscle mass, kg			
High [>23, *n* = 41]	Ref.		
Middle [20–23, *n* = 43]	1.068	0.434–2.629	0.886
Low [<20, *n* = 44]	2.382	1.083–5.242	0.031
Body fat, kg			
High [>16, *n* = 43]	Ref.		
Middle [11.5–16, *n* = 42]	2.565	1.045–6.300	0.040
Low [<11.5, *n* = 43]	3.068	1.270–7.409	0.013
Body fat percentage, %			
High [>29, *n* = 42]	Ref.		
Middle [22.5–29, *n* = 40]	2.545	1.037–6.246	0.041
Low [<22.5, *n* = 46]	2.609	1.081–6.296	0.033
Abdominal fat ratio			
High [>0.88, *n* = 51]	Ref.		
Middle [0.84–0.88, *n* = 39]	2.342	1.024–5.356	0.044
Low [<0.84, *n* = 38]	2.987	1.302–6.851	0.010

*HR*, hazard ratio; *CI*, confidence interval; *SGRQ*, St. George’s Respiratory Questionnaire; *CT*, computed tomography; *BMI*, body mass index; *FVC*, forced vital capacity; *FEV*_*1*_, forced expiratory volume in 1 s; *Ref*, reference

The final multiple logistic regression model showed that presence of cavities (adjusted hazard ratio [aHR], 4.577; 95% CI, 2.364–8.861), middle (aHR, 2.758; 95% CI; 1.112–6.843) or low tertiles (aHR, 3.084; 95% CI, 1.241–7.668) of abdominal fat ratio, and albumin level <3.5 g/dL (aHR, 12.943; 95% CI, 2.588–64.718) were associated with progression of NTM-LD (Table 5).

**Table 5:** Predictors of disease progression: multivariate analysis

	Adjusted HR	95% CI	*p*-value
Abdominal fat ratio			
High [>0.88, *n* = 51]	Ref.		
Middle [0.84–0.88, *n* = 39]	2.758	1.112–6.843	0.029
Low [<0.84, *n* = 38]	3.084	1.241–7.668	0.015
Albumin, g/dL			
≥ 3.5	Ref.		
< 3.5	12.943	2.588–64.718	0.002
Presence of cavity			
Absent	Ref.		
Present	4.577	2.364–8.861	<0.001

*HR*, hazard ratio; *CI*, confidence interval; *Ref*, reference

### Serum adipokine levels

Serum leptin and adiponectin levels were not different between patients with or without progression of NTM-LD. However, the leptin/adiponectin ratio (0.58 *vs.* 0.94 ng/μg, *p* = 0.089) was lower, and the leptin normalized to total body fat ratio was higher (0.60 *vs.* 0.53 ng/mL/kg, *p* = 0.086) among patients with progression, although statistical significance (*p* < 0.05) was not reached. The adiponectin normalized to total body fat ratio was higher among NTM-LD patients with progression (0.82 *vs.* 0.60 μg/mL/kg, *p* = 0.02) (Table 6).

**Table 6:** Comparison of adipokine serum levels at study entry in patients with or without disease progression

	Total patients (*N* = 147)	Progressed (*N* = 47)	Not progressed (*N* = 100)	*p*-value
Leptin, ng/mL	6.60 [4.20–11.70]	6.40 [3.60–10.70]	6.85 [4.33–12.33]	0.160
Adiponectin, μg/mL	9.58 [6.65–11.88]	10.10 [7.18–13.67]	9.27 [6.52–11.75]	0.337
Leptin/adiponectin ratio, ng/μg	0.76 [0.39–1.44]	0.58 [0.39–1.24]	0.94 [0.42–1.60]	0.089
Leptin/total body fat, ng/mL/kg	0.56 [0.40–0.75]	0.60 [0.43–0.82]	0.53 [0.37–0.72]	0.086
Adiponectin/total body fat, μg/mL/kg	0.72 [0.44–1.15]	0.82 [0.63–1.26]	0.60 [0.38–1.11]	0.020

Data are presented as median [interquartile range]

## Discussion

Through analysis of a cohort from a prospectively recruited, observational study, we showed that body composition (lower abdominal fat), radiographic feature (presence of cavity), and nutritional status (low serum albumin level) [21, 22] were associated with progression of NTM-LD.

Previous studies reported that presence of cavity [5] and fibrocavitary form [10, 23] of NTM-LD were associated with disease progression. Our study confirmed these previous observations and suggested that a higher bacterial burden in the cavity could be the reason for this association [24, 25].

Hypoalbuminemia is associated with increased complications and worse prognosis in various settings [22, 26–29]. Especially in patients with TB, a low albumin level was negatively associated with in-hospital death and 30-day survival [27]. Similarly, we demonstrated that hypoalbuminemia was associated with progression of NTM-LD. Hypoalbuminemia could be the result of the combined effects of inflammation and malnutrition. A vicious cascade of events ensues in which inflammation induces anorexia and reduces the effective use of dietary protein as well as augmenting the catabolism of the key somatic protein, albumin [30]. Therefore, a low albumin level reflects severe inflammation, which may affect disease progression.

Our study also showed that lower abdominal fat is associated with the progression of NTM-LD. In fact, previous studies reported that thinner chest subcutaneous fat thickness or low BMI were a risk factor for radiologic deterioration in MAC lung disease [9, 31]. Although disease progression could cause loss of fat, the fact that self-reported BMI before diagnosis of NTM-LD was lower among patients than in the general population [11] suggests that a lower amount of fat could be a risk factor for disease progression.

It has been recognized that adipose tissue participates actively in inflammation and immunity, producing and releasing a variety of pro-and anti-inflammatory factors including adipokines, such as leptin and adiponectin [32]. Previous studies reported higher serum levels of adiponectin [33] or adiponectin normalized for total body fat [12] among patients with NTM-LD than in healthy controls. Our data also showed increased serum levels of adiponectin normalized for total body fat and decreased leptin/adiponectin ratios among patients in whom NTM-LD progressed than in patients without progression. Previous reports and our study results suggest that a lower amount of fat, possibly associated with dysregulation of adipokines, may affect the development and progression of NTM-LD. The precise mechanism of these associations should be assessed in future studies.

Our study has some limitations. First, there is no global consensus regarding the definition of NTM-LD progression. One study defined the progression of NTM-LD as initiation of treatment [10], while another defined it as radiologic deterioration [9]. We defined the progression of NTM-LD as treatment required for NTM-LD by duty physicians. This could be a potential confounder because physicians might be more likely to initiate treatment for patients who are younger or patients with MAC rather than MABC lung disease. Second, we did not perform subgroup analysis according to each of the NTM species because of the small number of patients in each group. Differences in terms of predictors for disease progression could exist among patients with NTM-LD from different species. Lastly, because we do not know the exact timing of NTM infection in an individual patient, the risk factors for progression elucidated could be the results of disease progression.

## Conclusions

Progression of NTM-LD is associated with body composition in addition to presence of cavity and hypoalbuminemia. Lower abdominal fat ratio is an independent predictor of NTM-LD progression.

## Abbreviations

BMI: Body mass index
CI: Confidence interval
COPD: Chronic obstructive pulmonary disease
CT: Computed tomography
DLCO: Diffusing capacity for carbon monoxide
FEV_1_: Forced expiratory volume in 1 s
FVC: Forced vital capacity
HR: Hazard ratio
IQR: Interquartile range
MAC: *Mycobacterium avium* complex
NTM-LD: Nontuberculous mycobacterial lung disease
SGRQ: St. George’s Respiratory Questionnaire
